# Digital health and Buddhist network philosophy

**DOI:** 10.1192/bjb.2025.10144

**Published:** 2026-06

**Authors:** Shisei Tei, Junya Fujino, Tomomi Noda, Toshiya Murai

**Affiliations:** 1 Department of Psychiatry, Kyoto University, Kyoto, Japan; 2 Department of Psychiatry and Behavioral Sciences, Institute of Science Tokyo, Tokyo, Japan

**Keywords:** Communication, digital, network, flexibility, attention

## Abstract

Heated online communication reveals global challenges in the digital age, often fuelled by collective outrage. This article investigates how Buddhist network perspectives, paralleling digital reality, can inform mental health. Avatamsaka philosophy provides practical ways to navigate web complexities, suggesting that individual actions ripple across society. Recognising our interdependence and the impermanence of social responses deepens understanding of communication’s broader impact and dynamic interconnected worldviews. These perspectives support relational balance and cognitive flexibility, essential for alleviating online distress and conflicts, including acceptance of present circumstances and fostering motivation for positive change. Valuing connectedness while respecting individuality helps cultivate resilience, enriching therapeutic practices.

Stimulation of online content has increasingly contributed to real-world mental health concerns. In January 2025, the Aschaffenburg stabbings – where children were attacked in a park, resulting in two deaths – sparked public outrage and political debate in Germany. Similarly, the 2024 UK riots were fuelled by misinformation and transient narratives. Both events indicate how quickly collective emotions can surge, fade and resurface through digital platforms. These shifts reflect the transient and shared nature of our mind through which realities are co-created across digital and interpersonal spaces. Alongside these incidents, ongoing challenges, such as cyberbullying and echo chambers,^1–3^ have been associated with rising anxiety, depression and trauma. These effects deepen social divides and highlight the need for clinicians to engage broader value systems and context-sensitive care.

Digital technology has reshaped communication, extending social ties beyond family and local circles to distant and unknown individuals. Traditionally, philosophical and religious teachings nurture societal harmony; however, online interactions have reformed perceptions of individual and collective identity, making the self more dynamic yet also often ambiguous and fragile.^4,5^ Professional organisations call for greater focus on digital communication’s mental health impact.^2^

This article explores an adaptive approach to digital health, inspired by networked concepts in Buddhist Avatamsaka. The philosophy adopts the metaphor of Indra’s net (Table 1), a cosmic web of infinite interconnectedness where each node reflects and influences all others.^6^ Both systems exert interrelated effects: changes in a single node can affect the entire network, with nodes (e.g. individuals or websites) forming intricate interaction patterns. This view provides a practical framework for understanding how personal experiences shape, and are shaped, by online networks, thereby influencing public well-being. Beyond modelling digital systems, it can also serve as a grounding tool: in moments of digital distress, when outrage feels overwhelming, a brief pause to reflect on this web of mutual influence may soften reactivity and restore perspective. By applying Avatamsaka’s network view to digital reality, we outline potential therapeutic applications in three key areas: (a) networked dynamics, (b) reciprocity and (c) clinicians’ flexibility.

**Table 1:** Key concepts related to Buddhist philosophy

**Indra’s net**: A metaphor from the Avatamsaka Sutra that depicts the cosmos as a vast web of luminous jewels, each mirroring the others – symbolising infinite interdependence and mutual reflection, and fundamental equality. No element stands alone or exceeds another, illustrating the dynamic unity and co-arising of all phenomena. **Bodhisattva path**: A journey of compassion and wisdom aimed at relieving suffering while pursuing enlightenment. This perspective can help therapists develop attunement and reciprocal engagement in both online and offline interactions.

## Interconnected and individual perspectives in Avatamsaka

Among the various Buddhist traditions, Avatamsaka philosophy, originating in seventh-century China, is a key element of Mahayana Buddhism emphasising connectedness.^6,7^ While many Buddhist practices focus on individual liberation from suffering (e.g. mindfulness in Theravada),^8,9^ Avatamsaka uniquely describes selfhood and otherness as unified parts of a whole, in which each phenomenon reflects the other. Influencing schools such as Zen and Yogacara, it illuminates mutual relationships and the dynamic interplay of harmony and discord.^6,7^

This philosophy posits that our interconnected and individual natures, although seemingly contrasting, complement each other and support adaptive responses to social experiences. Its principle of dependent arising (*pratītyasamutpāda*) imbues life with multiple meanings, created through continuous encounters, while acknowledging the impermanence and selflessness (*anattā*) of all temporary connections. These perspectives are particularly useful when navigating the complex and distributed nature of online interactions, where actions ripple through networks.

According to the Middle Path in Buddhist thought, identity may be understood as neither purely fixed nor entirely fluid, but semi-solid, emerging through reciprocal influence and responsive to disruption and continuity.^6,7^ In psychiatric contexts, identity often appears rigid and enduring while in other settings, such as online environments, it may soften or shift in response to changing roles and relational inputs. This non-dual stance avoids extremes of rigidity or formlessness, suggesting that no state of being is inherently superior. It offers a clinical view of selfhood that is sufficiently stable to persist yet flexible enough to adapt, mirroring the evolving web of relational processes that shape and sustain experience.^10–13^

The Avatamsaka Sutra, a core Mahayana scripture, distils these principles into a vision of reality as an interpenetrating whole without fixed boundaries. Its portrayal of existence as a dynamic web supports a flexible view of identity, encouraging openness, awareness and responsiveness in both clinicians and clients. This worldview contrasts with the isolating effects of echo chambers and algorithmic filter bubbles, which fragment communication and reinforce division. The Sutra features intuitive, reciprocal understanding rooted in reflective wisdom rather than dogma or surface-level common sense. The following section investigates how these theories relate to practical challenges in digital communication.

## Sutra’s network theory and digital communication

The Sutra provides a framework for enhanced understanding of the structure and impact of digital communication. Complementing internet network theory, this Buddhist perspective offers insights into online misalignments, intergroup conflicts and distress, highlighting how virtual connections can both facilitate and distort communication.^1–3,14^

Paralleling this view of network formation and reciprocal impact, internet network theory examines how social tie strength and group dynamics shape complex online interactions and information flow. These dynamics create environments where relationships shift between localised and global interactions, ranging from tightly knit communities to expansive, loosely connected systems.^14^ Stronger ties often provide emotional validation, while weaker ties broaden informational horizons but may reduce personal exchanges, hinder active discernment and amplify misinformation.^1–3,14^

The Sutra’s perspective on interconnectedness helps clarify how online separation may trigger behaviours not typically expressed in person, often without recognising the pervasive and lasting effects. This viewpoint is also relevant to understanding the psychological influence of risky content sharing and the permanence of digital material.^1–3^

Research suggests that the Sutra’s emphasis on relational connection can enhance well-being, with Buddhist-informed social models linked to lower anxiety, depression and stigma, as well as improved social cognition.^15–17^ This relational view of online interconnection warrants further exploration to inform digital health interventions and promote more mindful online engagement.

## Therapeutic application and conceptual framework

The application of Avatamsaka’s interconnected perspective can enrich psychotherapy, especially in digital mental health (Table 2). The key concepts – (a) networked dynamics, (b) reciprocity and (c) clinicians’ flexibility – can be reinterpreted to address how online communication links, polarises and influences individuals. The philosophy provides a balanced perspective on collective interconnectedness and individual autonomy. This equips practitioners with a fresh approach for navigating complex clinical issues and digital affordances.

**Table 2:** The Sutra’s therapeutic value in digital relationships

**Networked dynamics** Avatamsaka’s network view parallels the digital life in which each action ripples across interconnected nodes. Applied alongside individual-focused care, it helps clinicians address problematic online behaviours. It may reveal overlooked or misjudged connections – such as the emotional impact of hate speech, unnoticed family support or hidden dependencies – that shape digital distress. Clinicians can guide clients to reflect on how their experiences are embedded in wider webs of interaction, shifting the focus from isolated symptoms to broader patterns. This may promote digital awareness and encourage clients to see themselves as shaped by – and actively shaping – their presence across digital and face-to-face interactions. **Reciprocity** Digital communication often lacks clear cues, requiring new ways of understanding relational dynamics. The Sutra’s emphasis on ‘exchanging self and other’ can offer therapists a method to restore empathic balance in one-sided or depersonalised narratives. Clinicians can help clients consider how their words and actions may be perceived – particularly when they feel misunderstood, alienated or overwhelmed by anonymous or artificial intelligence-mediated exchanges. By rehearsing emotionally grounded responses in imagined exchanges, clients can re-engage with clearer social awareness. For clinicians, cultivating this reflective posture also supports self-care and reduces burnout, consistent with the Sutra’s ideal of compassionate engagement while maintaining well-being. **Clinicians’ flexibility** The Middle Path encourages navigation between extremes – such as overinvolvement and disengagement – through a responsive and flexible orientation. It supports resilience and enables practitioners to stay attuned to changing clinical dynamics without becoming overly reactive or withdrawn. Rather than relying on fixed techniques, this process promotes openness to uncertainty and complexity. It also helps clients sit with emotional tension, confront perceived injustice or process grief – particularly in unpredictable digital settings. By cultivating flexibility and sensitive attunement to behavioural and communicative disruptions, this approach can support more tailored and sustainable care.

The following vignettes show how these concepts can enhance therapeutic strategies through real-world examples. In each case, Buddhist-informed ideas were not presented as doctrine, but rather used as reflective tools to support emotional regulation, social awareness and adaptive meaning-making. All participants provided written informed consent, and confidentiality was ensured by anonymisation of identifying details.

### Networked dynamics

The Sutra’s network dynamics regard connectedness and individuality as essential elements, helping clients cultivate adaptability. While virtual connectivity can alleviate isolation and foster community, it may also intensify social comparison, peer pressure and online harassment.^1–3,10^ Viewing social life through this lens of interconnectedness^7,18–20^ deepens understanding of solitude and personal struggles in online contexts. It also provides a way to acknowledging individuality within interconnection – recognising that even tight bonds may be provisional, while meaningful insight can arise through ordinary encounters.

#### Vignette 1

A young female university student (Y.) experienced social anxiety and loneliness. Her social media use, centred on Fitspiration (fitness and diet motivation), gradually amplified self-doubt as she frequently compared herself to idealised digital personas promoting unrealistic body standards. After questioning and criticising a movie actress’s diet-related content, she was overwhelmed by inconsiderate responses in a group chat.

The therapist used the concept of interconnectedness to explain how online communication can amplify negativity but also create space for support. This helped her recognise the emotional impact of online exchanges on self-image. Through relational mapping, the therapist helped identify overlooked sources of support (e.g. a trusted friend). Guided by this approach, she re-evaluated her self-image and strengthened supportive ties with helpful peers, establishing digital boundaries to disengage from harmful comparisons and limit exposure to triggering content.

#### Therapeutic focus

- Helped uncover relational patterns (e.g. how group dynamics shape self-perception);
- promoted the relational balance between social depth and diversity (e.g. friends and strangers); and
- aided engagement with positive nodes in the network and healthy distancing.

### Reciprocity

Online interactions involve complex implicit and explicit communications, and Sutra’s perspective on reciprocity can help guide clients through distress in digital contexts. The Bodhisattva path of Avatamsaka (Table 1) highlights reciprocity and the value of ‘exchanging self and other’, mentally placing oneself in another’s position to understand their perspective. This reflects Avatamsaka’s selfless orientation and its view of the Suchness of Reality (*tathatā*) as an undivided, interdependent nature of existence.^11^ In therapeutic settings, this orientation may serve as a subtle prompt for attunement and reciprocal awareness. It may provide a gentle cue for cultivating clinical responsibility and altruism.^18,20^ Furthermore, it resonates with the mentalising approach in psychotherapy and extends to online settings, where broader perspective-taking is framed by the ideas of Indra’s net and a non-dual view of relational selfhood, which supports both therapists and clients in navigating the emotional ripple effects of internet communication and the evolving experience of self shaped by online feedback.

#### Vignette 2

A man in his thirties (H.), working in sales, experienced stress and lack of fulfilment, mainly stemming from hybrid online meetings. Despite some work successes, his brief email responses left him feeling undervalued. Limited but negative anonymous feedback from customers further distorted his sense of acceptance and heightened self-doubt, alienating him in digital settings.

The therapist applied the concept of reciprocity to help H. distinguish between enduring personal values and transient digital adversity, such as reactions from customers and supervisors. This helped him adopt a more grounded stance in online interactions. By recognising past mutual appreciation and simulating emotionally tangible responses, the client practised a more adaptive engagement. This included expressing gratitude to colleagues and customers while nurturing mutual respect. Moreover, the therapist employed reciprocity as a tool for empathic connection, validating the client’s distress. Practical strategies included adjusting perspective and addressing compassion fatigue when relevant.

#### Therapeutic focus

- Promoted recognition of reciprocal interactions both in authentic and digital environments;
- advanced mutual communication and adjusted the sense of belonging; and
- built confidence in initiating positive interactions.

### Clinicians’ flexibility

Clinicians can navigate digital health by nurturing cognitive flexibility inspired by the Middle Path (*madhyampratipada*).^18^ This approach encourages a flexible, non-dual perspective between attachment and detachment, aiding the resolution of conflicts in the virtual environment (Table 2). The principle of *Anitya*, or the impermanence, highlights the fluid nature of relationships, supporting present-moment awareness and tolerance of uncertainty.^6,7^ This view nurtures resilience by cultivating acceptance of current circumstances while motivating constructive change. Without relying on technical or doctrinal terms, it offers clinicians a way to help patients gently reflect on the weight and reality of interconnectedness and impermanence – not as abstract or opposing ideas but as lived experiences, where no feeling exists in full isolation and all things, including suffering, are subject to change. This parallels dialectical behaviour therapy’s notion of ‘holding two truths’ – validating pain while promoting change – guiding clinicians in navigating relational or digital challenges with flexibility and responsiveness.

#### Vignette 3

A woman in her twenties (M.), working in web design, experienced maladaptive grief after the sudden death of her younger brother in a natural disaster. The grief was aggravated by excessive reliance on artificial intelligence-powered chat tools, which became her daily activity. Over time, rigid virtual engagement limited opportunities for genuine social communication. While chatting online occasionally alleviated her self-blame, it contributed to the development of depression and insomnia.

The clinician validated M.’s need for connection to her deceased brother and supported her in navigating the tension between emotional closeness and letting go, valuing stability while accepting life’s ambiguity. In addition, she explored how digital communication might subtly reshape aspects of self-perception or relational stance, and how overreliance on current artificial intelligence tools could complicate grief by reinforcing passivity or creating unrealistic expectations regarding relationships and support. This process facilitated a shift towards more durable coping strategies, such as sharing her bereavement with family and support groups. The clinician recognised synchrony and dissonance in M.’s online interactions as part of the evolving dynamics of human–artificial intelligence relationships. By addressing cognitive distortions and reframing distress, conflict and self-blame as natural components of grief, the clinician encouraged psychological flexibility. Grounded in the Middle Path, this approach supported a balanced engagement with uncertainty – helping M. remain present in ongoing relationships (e.g. family and work) without being overwhelmed by guilt linked to her younger brother.

#### Therapeutic focus

- Emphasised digital resilience;
- balanced acceptance and change within relationships; and
- developed a nuanced understanding of maladaptive online behaviours.

## Shifting attitudes and navigating online engagement

The Sutra’s connected view can aid in shifting attitudes to navigate online discord. Viewing digital engagement as part of a larger relational web that is dynamic and provisional helps shift focus away from emotionally charged content. It can potentially mitigate social anxiety, depression and impulsive or addictive behaviours^20^ associated with digital media use. This perspective illuminates the transient yet pervasive nature of virtual communication, highlighting mutual accountability alongside information exchange and goal-oriented transactions.

Tackling mental suffering in virtual communication benefits from balancing individual experience with broader responsibility – acknowledging connectedness, social obligation and cosmopolitan inclusivity^11^ in support of personal and public well-being. Avatamsaka philosophy presents a vision of shared reflection within a vast network,^6^ comparable to the complexity of digital networks. Online platforms often promote the notion that life should appear ordered and purposeful, shaping how people perceive themselves and relate to others.

The Sutra’s networked approach addresses the tendency to prioritise instant information transmission and immediate or self-centred gratification, rather than deliberate, thoughtful interactions. It encourages an adaptive acknowledgment of uncertainty and diversity, which may contribute to a more nuanced and sustainable digital engagement. Such flexibility enhances resilience to unexpected responses and non-linear benefits, fostering long-term social and reciprocal value over short-term rewards.

These perspectives envision individuality as emerging through nested relationships, shaped by ongoing interactions while forming a unified whole.^6^ They emphasise our simultaneous interdependence and self-direction, suggesting that being in the world – including virtual spaces – involves creating cohesive experiences and living socially and authentically, rather than anonymously. This view implies that we are not ultimate controllers of existence, but rather caretakers and active participants, echoing Heidegger’s concept of ‘Shepherds of Being’.

Examining communication through the lens of Avatamsaka philosophy and network theory provides a helpful framework for addressing the complexities of digital health. Recognising our interdependent nature while balancing relational depth and diversity can enhance therapeutic approaches. By respecting the sensitivity of connectedness and individuality, practitioners may better navigate online discord and attune to evolving interpersonal patterns. Change and acceptance permeate the digital sphere, oscillating between synchrony and dissonance, akin to the intricate dance of human precariousness. This perspective promotes a shift in mindsets to align with digitally shaped worldviews: many relationships are dynamic and provisional, whereas overlooked connections and incidental encounters may carry unexpected meanings. Moreover, these experiences, seen within a broader narrative, may not be entirely true or false but help us make sense of ourselves. Rather than chasing immediacy or certainty, we can transform digital challenges into opportunities for more intentional, flexible engagement. Bridging ancient insights with modern technology offers a way to cultivate resilience and intercorporeality: shared narratives and embodied experiences that can enrich digital health. Future research should examine additional case material to evaluate the clinical relevance of these perspectives across diverse settings.

## Data Availability

Data sharing is not applicable to this article because no data-sets were generated or analysed.
